# An Insight into Giant Cell Arteritis Pathogenesis: Evidence for Oxidative Stress and SIRT1 Downregulation

**DOI:** 10.3390/antiox10060885

**Published:** 2021-05-31

**Authors:** Alessandro Ianni, Poonam Kumari, Shahriar Tarighi, Flavia Rita Argento, Eleonora Fini, Giacomo Emmi, Alessandra Bettiol, Thomas Braun, Domenico Prisco, Claudia Fiorillo, Matteo Becatti

**Affiliations:** 1 Department of Cardiac Development and Remodeling, Max-Planck-Institute for Heart and Lung Research, Ludwigstrasse 43, 61231 Bad Nauheim, Germany; alessandro.ianni@mpi-bn.mpg.de (A.I.); Poonam.Kumari@mpi-bn.mpg.de (P.K.); Shahriar.Tarighi@mpi-bn.mpg.de (S.T.); Thomas.Braun@mpi-bn.mpg.de (T.B.); 2 Department of Experimental and Clinical Biomedical Sciences “Mario Serio”, University of Firenze, 50134 Firenze, Italy; flaviarita.argento@unifi.it (F.R.A.); eleonora.fini@unifi.it (E.F.); matteo.becatti@unifi.it (M.B.); 3 Department of Experimental and Clinical Medicine, University of Firenze, 50134 Firenze, Italy; giacomo.emmi@unifi.it (G.E.); alessandra.bettiol@unifi.it (A.B.); domenico.prisco@unifi.it (D.P.)

**Keywords:** giant cell arteritis (GCA), oxidative stress, SIRT1

## Abstract

Giant cell arteritis (GCA), medium and large vessel granulomatous vasculitis affecting the elderly, is characterized by a multitude of vascular complications, including venous thrombosis, myocardial infraction and stroke. The formation of granulomatous infiltrates and the enhanced accumulation of proinflammatory cytokines are typical features of this condition. The GCA pathogenesis remains largely unknown, but recent studies have suggested the involvement of oxidative stress, mainly sustained by an enhanced reactive oxygen species (ROS) production by immature neutrophils. On this basis, in the present study, we intended to evaluate, in GCA patients, the presence of systemic oxidative stress and possible alterations in the expression level of nuclear sirtuins, enzymes involved in the inhibition of inflammation and oxidative stress. Thirty GCA patients were included in the study and compared to 30 healthy controls in terms of leukocyte ROS production, oxidative stress and SIRT1 expression. Our results clearly indicated a significant increase (*p* < 0.05) both in the ROS levels in the leukocyte fractions and plasma oxidative stress markers (lipid peroxidation and total antioxidant capacity) in the GCA patients compared to the healthy controls. In PBMCs from the GCA patients, a significant decrease in SIRT1 expression (*p* < 0.05) but not in SIRT6 and SIRT7 expression was found. Taken together, our preliminary findings indicate that, in GCA patients, plasma oxidative stress is paralleled by a reduced SIRT1 expression in PBMC. Further studies are needed to highlight if and how these alterations contribute to GCA pathogenesis.

## 1. Introduction

Giant cell arteritis (GCA; formerly Horton disease or temporal arteritis) is a systemic vasculitis derived from the granulomatous inflammation of medium to large-sized vessels [1]. GCA concerns one or more branches of the carotid arteries, especially the temporary artery, although other vessels may be affected as well [2]. The associated symptoms include temporal headache and scalp tenderness, scalp infraction and other ischemic presentations. Optic neuropathy represents the most common complication of GCA, and delayed treatment can lead to sight loss. Untreated GCA may cause cardiovascular complications as well, including venous thrombosis, myocardial infraction and stroke [1,3,4,5]. The incidence of the disease is higher in people aged 70 or older, indicating that aging is one of the main factors that predisposes one to GCA [1].

Dendritic cells (DCs) residing in the vessels play a key role in GCA pathogenesis. Activated DCs promote the robust recruitment of lymphocytes and macrophages to the vessels, leading to the formation of granulomatous infiltrates, which includes multinucleated giant cells that are derived from the fusion of highly activated macrophages [1,6]. The enhanced accumulation of proinflammatory cytokines such as interferon gamma (IFN-γ) and interleukin 17 (IL-17), especially secreted by CD4 T cells and by activated macrophages, is a typical feature of GCA [1]. The secretion of these factors plays a pivotal role in vascular remodeling by promoting the aberrant activation of fibroblasts, proliferation of smooth muscle cells and activation of endothelial cells, leading to intimal hyperplasia and vascular occlusion [1,6].

As regards GCA pathogenesis, recent studies have suggested the involvement of oxidative stress, mainly sustained by an enhanced reactive oxygen species (ROS) production from immature neutrophils. However, the precise mechanisms underlining this process still remain largely uncharacterized [4].

In this context, our group recently showed in another vasculitis (Bechet disease (BD)) that neutrophil activation contributes to thrombus formation through ROS-mediated mechanisms, suggesting a potential similar process in GCA [7].

Sirtuins are greatly conserved enzymes principally acting as NAD^+^-dependent protein deacetylases and mono-ADP ribosyltransferase enzymes [8]. In mammals, sirtuins build a family of seven members (SIRT1–SIRT7) sharing a conserved catalytic domain but differing in N- and C-terminal domains, which are crucial for their subcellular localization and interactions with specific targets. Sirtuins show a broad subcellular distribution: SIRT1, SIRT6 and SIRT7 are mostly present in the nucleus, and SIRT2 is located in the cytoplasm, while SIRT3, SIRT4 and SIRT5 are mitochondrial enzymes [8]. Interestingly, nuclear Sirtuins SIRT1, SIRT6 and SIRT7 have been shown to play critical roles in controlling oxidative stress and inflammation [9,10,11]. SIRT1 is the best-characterized member of the family and acts as a key inhibitor of inflammation and oxidative stress [12]. This function is mediated by the SIRT1-dependent deacetylation of the critical transcription factors such as NF-κB [13,14], GATA-3 [15], FOXO3A, p53 and PGC-1α [9].

Based on this background, the aim of the present study was to evaluate, in GCA patients, the occurrence of systemic oxidative stress and the possible alterations in the expression level of nuclear sirtuins in the PBMC fraction to gain insight into the GCA pathogenetic mechanisms.

## 2. Material and Methods

### 2.1. Participants

This cross-sectional study included 30 consecutive patients with GCA who attended the Vasculitis Centre of Careggi University Hospital (Florence, Italy) and 30 age and sex-matched healthy control subjects. Patients were diagnosed as having GCA disease according to the ACR criteria [5] and were receiving corticosteroid therapy. Patients with other autoimmune diseases/vasculitis, infectious or neoplastic conditions were excluded.

This study conformed to the principles outlined in the Declaration of Helsinki. The study protocol was approved by the local ethics committee, and all the subjects signed an informed written consent before entering the study.

### 2.2. Sample Collection

Blood was collected in vacutainer tubes containing 0.109-mol/L buffered trisodium citrate (1:10) or ethylenediamine tetraacetic acid (EDTA 0.17 mol/L). Sodium citrate plasma was obtained after centrifugation (1500× *g* for 15 min at 4 °C), and aliquots were immediately used for experiments or stored at −80 °C for additional analyses.

### 2.3. Isolation of Peripheral Blood Mononuclear Cells (PBMCs)

For this analysis, fresh peripheral EDTA anticoagulated venous blood (5 mL) was used. PBMCs obtained by density gradient centrifugation using Ficoll-Paqueplus (DAKEWE Biotech, Beijing, China) according to the manufacturer’s instructions were further resuspended in red blood cell lysis buffer for 5 min and ultimately collected by centrifugation.

### 2.4. Blood Leukocytes Intracellular ROS Levels

As previously described by our group [7,16,17], 100 µL of EDTA anticoagulated blood resuspended in 2 mL of BD FACS Lysing Solution (Becton Dickinson Biosciences, San Jose, CA, USA) were mixed and incubated at room temperature in the dark for 15 min, followed by centrifugation at 700× *g* for 7 min at 20 °C. The supernatant was immediately discarded, and the cells were then washed twice in Phosphate Buffered Saline (PBS). Intracellular ROS levels were assayed after the cells were incubated with 2’,7’-dichlorodihydrofluorescein diacetate (H_2_DCF-DA, 2.5 µM) for 30 min at 37 °C (Invitrogen, Carlsbad, CA, USA) in RPMI medium without serum and phenol red. After washing and resuspension in PBS, cells were analyzed using a FACSCanto flow cytometer (Becton-Dickinson, San Jose, CA, USA) with a sample flow rate adjusted to about 1000 cells/s. Gates were defined using the specific forward-scatter and side-scatter properties of the individual cell populations. Cell viability was checked by flow cytometry with propidium iodide staining and was found to exceed 95%. Data were analyzed using BD FACSDiva software (version 8.0, Becton-Dickinson, San Jose, CA, USA).

### 2.5. Plasma Lipid Peroxidation Estimation (Thiobarbituric Acid Reactive Substances Assay; TBARS Assay)

Plasma Thiobarbituric Acid Reactive Substances (TBARS) were assessed by a TBARS Assay Kit (Cayman Chemical, Ann Arbor, MI, USA) following the manufacturer’s instructions. This assay was based on the reaction (performed for 1 h at 95 °C) of thiobarbituric acid with lipoperoxidation products, which resulted in the formation of a chromophore adduct estimated spectrofluorometrically with excitation at 530 nm and emission at 550 nm on a microplate fluorometer (Biotek Synergy H1, Winooski, VT, USA). Results were expressed as malondialdehyde (MDA) (nmol/mL) [18].

### 2.6. Plasma Total Antioxidant Capacity Assay (Oxygen Radical Absorbance Capacity Assay; ORAC Assay)

ORAC assay (Oxygen Radical Absorbance Capacity) was founded on the fluorescence decay of the probe fluorescein upon oxidation by peroxyl radicals generated by the thermal decomposition of azo compounds such as 2,2′-azobis(2-amidinopropane) dihydrochloride (AAPH). A 6-nM fluorescein solution in 75-mM sodium phosphate buffer (pH 7.4) and 250-µM Trolox (as a standard), a water-soluble analog of vitamin E, were used. After a preincubation of 70 µL of each sample for 30 min at 37 °C in each well with 100 µL of fluorescein, an AAPH solution (19-mM final concentration) was added to start the reaction. Fluorescence was measured setting the excitation at 485 nm and emission at 537 nm on a microplate fluorometer (Biotek Synergy H1, Winooski, USA). Results were expressed as Trolox equivalents (µM) and then normalized for the protein concentration [19].

### 2.7. Oxidative Stress Index (OSI)

Oxidative Stress Index (OSI) was calculated as the Plasma lipid peroxidation/Plasma Total Antioxidant Capacity × 100 [20].

### 2.8. Western Blot Analysis

Western blot analysis was performed as already described [21]. The following antibodies were used in this study: SIRT1 (8469; Cell Signaling Technology, Danvers, MA, USA), SIRT7 (5360; Cell Signaling Technology, Danvers, MA, USA), SIRT6 (12486; Cell Signaling Technology, Danvers, MA, USA), GAPDH (2118; Cell Signaling Technology, Danvers, MA, USA), and actin (A2103; Sigma-Aldrich, St. Louis, MO, USA).

### 2.9. Statistical Analysis

All the experiments were performed in triplicate at 3 different time points. For each time point and for each subject, the mean value of the three replicates was calculated. The group median and interquartile range were calculated considering the mean values for each subject as single values in the calculations. The Mann–Whitney test was applied to compare the considered parameters between the GCA patients and healthy controls. All statistical operations data were processed using Graph Pad Prism 5 software (version 5.0, GraphPad Software, San Diego, CA, USA). A value of *p* < 0.05 was considered as statistically significant.

## 3. Results

### 3.1. Subjects

The demographic, clinical and therapeutic characteristics of the 30 GCA patients included in the study are reported in Table 1.

Most patients (21/30) were female, with a median age at diagnosis of 74 (69–80) years and at inclusion in the study of 76 (71–81) years.

The most common disease manifestations at diagnosis included headache (70%), arthralgia/myalgia (70%), jaw claudication (40%) and weight loss (36.7%).

Eleven patients also presented concomitant polymyalgia rheumatica.

Three patients had a history of previous cardiovascular events. Regarding the cardiovascular risk factors, 17 patients presented hypertension, 12 dyslipidemia and seven diabetes.

As for pharmacological therapy, twenty-six patients (86.7%) were receiving corticosteroids at a median daily dose of 10 (5–17.5) mg, and twelve patients were on active antiplatelet therapy.

### 3.2. Blood Leukocytes from GCA Display Increased Intracellular ROS Levels

**Figure 1:**
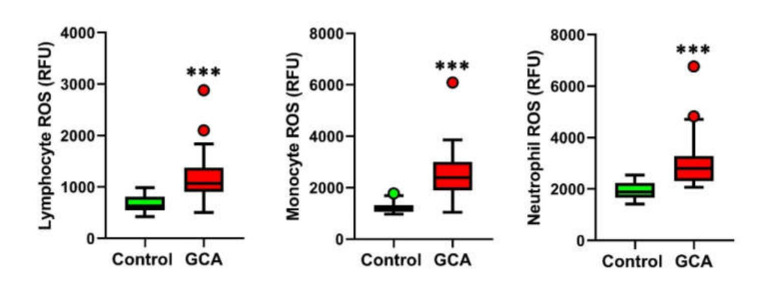
Blood leukocyte ROS production in the GCA patients and controls. Lymphocyte, monocyte and neutrophil ROS production in the GCA patients (*n* = 30) and controls (*n* = 30). *** *p* < 0.001.

The intracellular ROS levels in the blood leukocyte subpopulations of the lymphocytes, monocytes and neutrophils were measured in 30 GCA patients and 30 healthy subjects. As reported in Figure 1, the GCA patients showed a significant increase in the ROS levels in all the three leukocyte fractions as compared to the healthy controls.

### 3.3. Signs of Plasma Oxidative Stress in GCA

**Figure 2:**
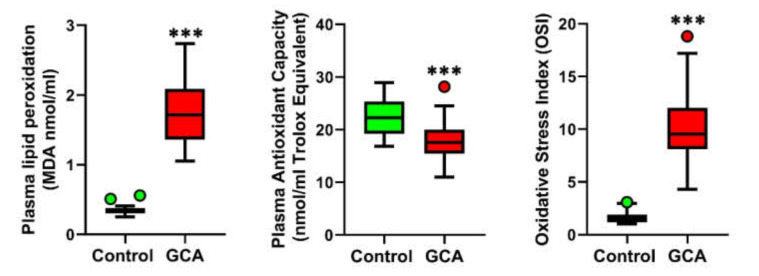
Plasma lipid peroxidation, plasma total antioxidant capacity and Oxidative Stress Index in the GCA patients (*n* = 30) and controls (*n* = 30). *** *p* < 0.0001.

In the GCA patients, oxidative stress evaluation in plasma samples revealed a significantly higher lipid peroxidation (0.34 (0.30–0.38) vs. 1.72 (1.36–2.09), *p* < 0.0001), a lower total antioxidant capacity (TAC) (22.29 (19.22–25.37) vs. 17.63 (15.47–20.01), *p* < 0.0001) and a higher Oxidative Stress Index (1.49 (1.19–1.92) vs. 9.54 (8.12–12.04), *p* < 0.0001) compared to the healthy controls, as shown in Figure 2.

### 3.4. PBMCs Derived from GCA Patients Display a Significant Decrease in SIRT1 Expression

**Figure 3:**
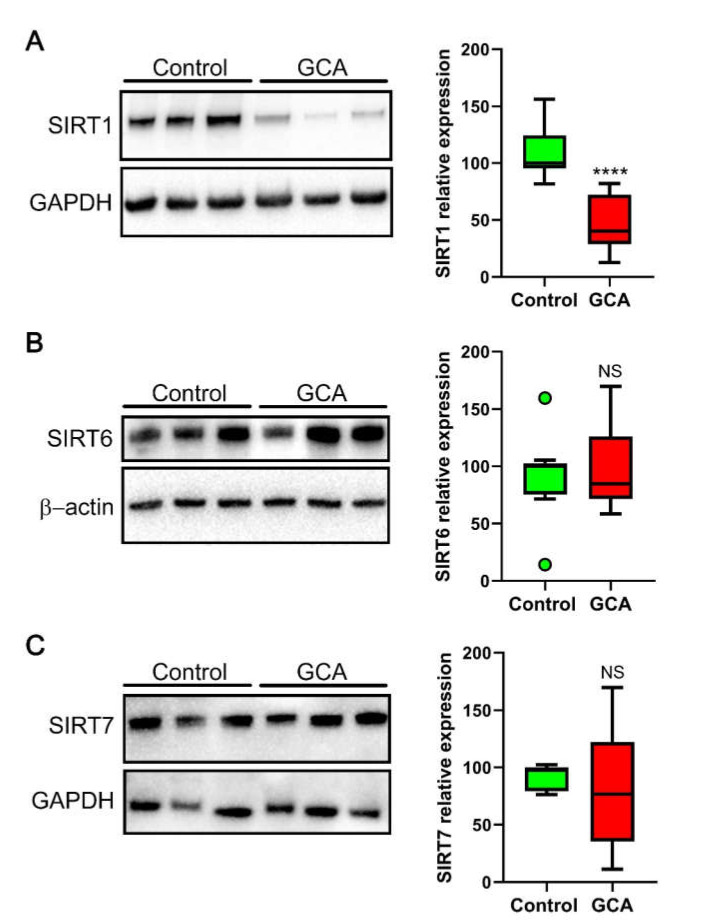
Western blot analysis of SIRT1 (**A**), SIRT6 (**B**) and SIRT7 (**C**) expression in PBMCs derived from the GCA patients and healthy controls. Quantification of sirtuins expression normalized to the relative loading control (GAPDH or Actin) ± SD is shown in the right histograms (*n* = 9; **** *p* < 0.0001; NS: not significant).

Nuclear sirtuins SIRT1, SIRT6 and SIRT7 play a pivotal role in controlling oxidative stress and inflammation [9,10,11]. Thus, we wanted to assess the expression of nuclear sirtuins in PBMCs derived from the controls and GCA patients. The Western blot analysis for SIRT1 expression revealed a significant downregulation of SIRT1 in the PBMCs derived from the GCA patients as compared to the healthy controls (Figure 3A). In sharp contrast, the expression of nuclear sirtuins SIRT6 and SIRT7 remained unaffected (Figure 3B,C).

### 3.5. Blood Oxidative Stress Parameters and Sirtuin Expression in GCA Patients with and without Diabetes

Oxidative stress, inflammation and sirtuin expression are closely related to the pathogenesis of insulin resistance and type 2 diabetes mellitus [22]. To assess whether diabetes acts as a confounding factor, our data were analyzed in GCA patients with and without diabetes (Table 2). No statistically significance was found between the two groups.

## 4. Discussion

GCA is a systemic vasculitis with a relatively high prevalence and a not negligible burden of disease in the elderly [23]. The correlation between GCA and aging is likely to arise from the progressive age-related deterioration of the immune system; this phenomenon, known as immunosenescence, can lead to an impairment in immunocompetent cells, forcing the host to activate alternative immune pathways that can cause chronic inflammatory tissue damage [24]. Additionally, age-associated changes in the blood vessels might contribute to the aberrant cross-talk between the immune system and vessels that is responsible for GCA. Notwithstanding, the exact mechanisms that facilitate GCA onset in the elderly population remain largely unknown [6,9].

The genetic risk factors have also been described, and the identified genes are connected to the immune system, underling the critical role of immunity in GCA pathogenesis [25]. Despite genetic predisposition, the altered expression of factors that can predispose GCA development may equally contribute to its pathogenesis. In this study, we investigated, for the first time in GCA patients, the occurrence of oxidative stress and sirtuin expression to gain insight into the pathogenetic mechanisms of the disease and, in particular, to its vascular complications.

Our findings show a robust downregulation of the expression of the histone/protein deacetylase SIRT1 in PBMCs derived from GCA patients as compared to healthy controls, paired by a remarkable increase in oxidative stress markers.

SIRT1 is known to be a critical antiaging molecule, and a decline in SIRT1 expression and activity during aging is correlated with severe disorders such as cardiovascular diseases, neurodegenerative disorders and cancer [26]. Thus, the decline in SIRT1 expression observed in our GCA cohort might, at least in part, explain the association between aging and the GCA pathogenesis.

SIRT1 has been recognized as a critical inhibitor of inflammatory responses and oxidative stress. The SIRT1-dependent deacetylation of the critical transcription factors such as p53, FOXO3A and PGC-1α and their consequent stimulation of the expression of the genes involved in antioxidant defenses dramatically contribute to the inhibition of oxidative stress [9]. Indeed, SIRT1 depletion in mouse macrophage cell lines greatly increases the expression of the lipopolysaccharide (LPS)-induced proinflammatory cytokines such as TNFα and IL-6 and IL-8 by activation of the transcription factor NF-κB, while, consistently, the pharmacological activation of SIRT1 suppresses the inflammatory response [13]. Similarly, SIRT1 suppresses the inflammatory response in an NF-κB-dependent manner in human monocyte cells treated with cigarette smoke extracts [27]. On this basis, we can suppose that, in GCA, the reduced SIRT1 expression might be directly responsible for the redox imbalance observed in PBMCs in our and other studies [4,28].

In particular, our findings indicate that neutrophil ROS production is particularly increased in GCA patients as compared to healthy controls. This result supports previous preclinical evidence showing that immature neutrophil-derived ROS production leads to enhanced protein oxidation and permeability of the endothelial barrier in vitro, thus potentially contributing to the pathogenesis of vascular complications in GCA [4].

Concomitantly, our data also show that lymphocyte and monocyte ROS production is enhanced in GCA. The presence of oxidative stress in GCA patients was confirmed by calculation of the OSI index, a reliable indicator of systemic redox dyshomeostasis. The DC-mediated recruitment and hyperactivation of lymphocytes and macrophages is known to be implicated in the formation of granulomatous infiltrates in GCA [1,6], and SIRT1 has been shown to play a direct regulatory role in suppressing these proinflammatory pathways [29].

It has been shown that the downregulation of sirtuin family members, such as SIRT1 and SIRT6, is closely related to the onset of insulin resistance and type 2 diabetes mellitus via inflammation and oxidative stress [30,31,32,33,34]. Indeed, in our study, diabetes, which was present in 23% of the enrolled GCA patients, could represent a confounding factor. However, our statistical analysis demonstrated that the sirtuin expression and oxidative stress parameters were not statistically different in the GCA patients with and without diabetes, confirming that SIRT1 expression is strictly linked to oxidative stress in GCA.

**Figure 4:**
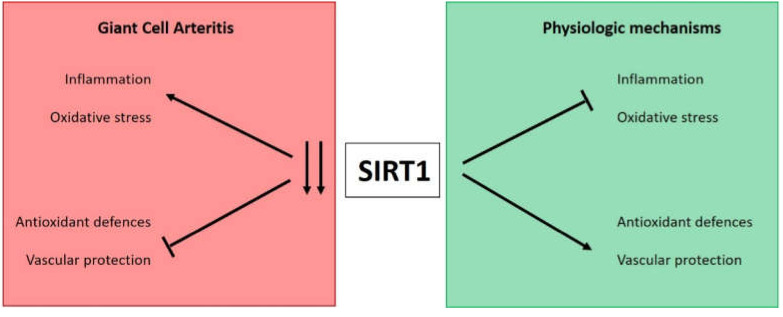
Schematic diagram illustrating the possible effects of SIRT1 downregulation in GCA.

Considering the reported findings, it could be speculated that a declined SIRT1 expression in GCA patients might be linked to the observed oxidative stress and that these phenomena could have a role in the pathogenesis of this vasculitis, although further research is warranted to support this claim (Figure 4).

## 5. Conclusions

The therapeutical approaches against GCA are currently based on the long-term use of glucocorticoids, which are, however, associated with some relevant safety concerns. Only recently, anti-IL6R tocilizumab proved its efficacy in GCA, although only around 55–85% patients achieved complete and long-term disease remission while on this treatment [35,36], and not all patients can successfully discontinue glucocorticoids [37]. Thus, the development of innovative therapies against GCA is imperative to improve the life quality of patients. The further characterization of SIRT1 functions in GCA may offer new opportunities in this direction, as SIRT1 activators are under evaluation in different clinical trials for the treatment of numerous human diseases [38,39].

## Figures and Tables

**Table 1:** Clinical characteristics of the GCA patients and matched healthy controls enrolled in the study.

Patients Features	GCA Cases (% out of 30)	Healthy Controls (% out of 30)
Age at diagnosis, years (median, IQR)	74 (69–80)	
Age at inclusion, years (median, IQR)	76 (71–81)	75 (71–82)
Disease duration, years (median, IQR)	1 (1–2)	-
**Gender**		
Female	21 (70%)	21 (70%)
Male	9 (30%)	9 (30%)
**Disease Manifestations at Diagnosis**		
Headache	21 (70%)	-
Arthralgia/myalgia	21 (70%)	
Jaw claudication	12 (40%)	
Weight loss	11 (36.7%)	
Cutaneous symptoms	9 (30%)	
Fever	8 (26.7%)	
Visual loss	6 (20%)	
Cardiovascular events	3 (10%)	
Polymyalgia rheumatica	11 (36.7%)	
**Cardiovascular Risk Factors**		
Hypertension	17 (56.7%)	0 (0%)
Dyslipidemia	12 (40%)	0 (0%)
Diabetes	7 (23.3%)	0 (0%)
Cardiopathy	3 (10%)	0 (0%)
Obesity	1 (3.3%)	0 (0%)
**Ongoing Therapy**		
Corticosteroids	26 (86.7%)	
Median prednisone daily dose, mg	10 (5–17.5)	
Antiplatelet therapy	12 (40%)	

**Table 2:** Blood oxidative stress parameters and sirtuin expression in the GCA patients with and without diabetes.

	GCA Patients with Diabetes (*n* = 7)	GCA Patients without Diabetes (*n* = 23)	*p*-Value *
**Oxidative Stress Parameters**			
Lymphocytes ROS (RFU)	1079 (984–1635)	1065 (877–1330)	0.447
Monocytes ROS (RFU)	2703 (2169–3321)	2369 (1881–2923)	0.447
Neutrophil ROS (RFU)	3029 (2362–3421)	2753 (2237–3217)	0.270
Plasma lipid peroxidation (MDA nmol/mL)	2.0 (1.5–2.3)	1.6 (1.4–2.1)	0.390
Plasma antioxidant capacity (nmol/mL Trolox Equivalent)	18.5 (15.5–23.8)	16.7 (15.5–19.6)	0.508
OSI (MDA/ORAC * 100)	11.3 (9.5–12.0)	9.1 (7.9–12.6)	0.391
**SIRT Relative Expression**			
SIRT1	52 (42–58)	42 (39–50)	0.134
SIRT6	134 (82–158)	85 (58–113)	0.292
SIRT7	111 (50–164)	76 (36–136)	0.249

* From the Mann–Whitney test for unpaired data.

## Data Availability

The data that support the findings of this study are available from the corresponding author upon reasonable request.
